# Isolation of a natural product with anti-mitotic activity from a toxic Canadian prairie plant

**DOI:** 10.1016/j.heliyon.2021.e07131

**Published:** 2021-05-24

**Authors:** Layla Molina, David E. Williams, Raymond J. Andersen, Roy M. Golsteyn

**Affiliations:** a Natural Product and Cancer Cell Laboratories, University of Lethbridge, Lethbridge, AB, T1K 3M4 Canada; b Department of Earth, Ocean, Atmospheric Sciences, University of British Columbia, Vancouver, BC, V6T 1Z4 Canada

**Keywords:** Asteraceae, Cdk1, *Hymenoxys richardsonii*, Hymenoratin, Mitotic spindle, Mitosis, Sesquiterpene lactone

## Abstract

We are investigating plants from the prairie ecological zone of Canada to identify natural products that inhibit mitosis in cancer cells. Investigation of plant parts from the Canadian plant species *Hymenoxys richardsonii* (Asteraceae) revealed that leaf extracts (PP-360A) had anti-mitotic activity on human cancer cell lines. Cells treated with leaf extracts acquired a rounded morphology, similar to that of cells in mitosis. We demonstrated that the rounded cells contained mitotic spindles and phospho-histone H3 using the techniques of immunofluorescence microscopy. By biology-guided fractionation of *H. richardsonii* leaves, we isolated a sesquiterpene lactone named hymenoratin, which had not been previously assigned a biological activity. Cells treated with hymenoratin have phospho-histone H3 positive chromosomes, a mitotic spindle, and enter a prolonged mitotic arrest in which the spindles become distorted. By Western blot analysis, hymenoratin treated cells acquire high levels of cyclin B and dephosphorylated Cdk1. There is a growing body of evidence that select members of the sesquiterpene lactone chemical family have anti-mitotic activity.

## 1 Introduction

The Asteraceae taxonomical family is recognized as a source of natural products known as sesquiterpene lactones, which may have roles in modifying animal herbivory [1, 2]. To date, the majority of sesquiterpene lactones that arrest cells in mitosis have been isolated from species within the Asteraceae family [3]. Many plants species that flourish in the prairie ecological zone are members of the Asteraceae family, of which the prototype is the sunflower. We reasoned that Asteraceae plants that are present on Canadian prairies and toxic to mammals might be sources of sesquiterpene lactones with antimitotic activity.

From more than 5000 sesquiterpene lactones described [4], only a handful have been reported to induce a mitotic arrest in cells [3]. Some of these, such as 6-O-angeloylplenolin induced a mitotic arrest possibly by inhibiting Skp1-Cullin-F-box protein (SCF) complex [5]. The sesquiterpene lactones psilostachyin A and C inhibit the G2/M checkpoint and arrest cells in mitosis with aberrant mitotic spindles [6]. One of the best described sesquiterpene lactones, parthenolide, arrests the cell cycle by inhibiting the tubulin carboxypeptidase [7]. The precise mechanism of action of anti-mitotic sesquiterpene lactones is not known, and it remains possible that they have more than one target.

We are investigating if plants in the prairie ecological zone of Canada contain natural products that inhibit mitosis or the cell cycle in cancer cells [8]. To understand better how a subgroup of sesquiterpene lactones induce mitotic arrest we investigated the *Hymenoxys richardsonii* species from the Asteraceae family [9]. *H. richardsonii,* commonly known as Colorado rubberweed or Richardson's bitterweed is native to prairie ecological zones in Canada [10]. The plant has 1-10 stems varying in length from 7–24 cm with leaves that are long, moderately pubescent, and simple or divided into 3–7 segments [11] (Supplemental Figure 1). It is toxic to livestock, and several chemicals have been isolated from it although none have been shown to have antimitotic activity when applied to cells [12]. By using a phenotypic assay approach, we report the identification and characterization of an anti-mitotic sesquiterpene lactone from *H. richardsonii*, which provides insight into structures from this chemical family and their associated biological activity in cancer cells.

## 2 Materials and methods

### 2.1 Plant material and collection

Permits from the provincial and local governments were acquired for collections. Aerial plant parts and roots of *H. richardsonii* were collected when flowering by sustainable practice in southern Alberta, Canada at 49°41′5 N and −112°52′2 W, elevation approximately 900 m during 2016 and 2017. Plant taxonomy was confirmed to species [9, 10, 13] and verified by the University of Lethbridge Herbarium. Detailed information about *Hymenoxys* taxonomy and distribution has been described [11]. A voucher specimen was provided to the Herbarium as #Golsteyn 360. Following harvest, plants were dried at room temperature and stored in the dark in paper bags in a dry environment at room temperature until extraction.

### 2.2 Preparation of plant extracts

Plant parts (flowers, leaves, roots) were separated and extracts were prepared from leaves by grinding dried material to a fine powder and stirring it overnight at room temperature in either 75% (v/v) ethanol in water (PP-360A) or in 100% dichloromethane (PP-360B). The suspensions were filtered, dried by exposure to forced air at room temperature, and stored in darkness at 21–23 °C until use. Yields of extract from dried plant were 4.4% for PP-360A and 4.1% for PP-360B. Samples were dissolved in dimethyl sulfoxide (DMSO) (Sigma-Aldrich; D2438) to concentrations of 50 mg/mL and tested. Extracts from flowers and from roots were also prepared in 75% (v/v) ethanol in water in a manner similar to that of leaves.

### 2.3 Bioassay-guided isolation of hymenoratin

The chemical fractionation was performed as follows: 25 g of dried plant material was extracted twice with 200 mL methanol overnight at room temperature. The combined methanol extracts were dried and partitioned between water (1:300) and ethyl acetate (3:75). These extracts were also combined and dried. Approximately 1/4 of the ethyl acetate soluble material was chromatographed on Sephadex LH20 using a 86 × 2.6 cm column with 80% (v/v) methanol/CH_2_Cl_2_ as eluent. The fractions were analyzed by the cell rounding assay using concentrations of 15 and 50 μg/mL. The bioactive fraction obtained was then purified by C-18 reverse-phase HPLC using an InertSustain 25 × 1 cm column with 80% (v/v) H_2_O/acetonitrile as eluent with a flow rate of 2 mL/min to give 26.8 mg of hymenoratin, comprising 0.4% of the total dried plant material. The structure of hymenoratin was confirmed by detailed analysis of standard 1D and 2D Nuclear Magnetic Resonance (NMR) spectra (see supplementary material) and Low-Resolution Electrospray Ionization Mass Spectrometry (LRESIMS). NMR spectra were recorded on a Bruker AV-600 spectrometer with a 5 mm CPTCI cryoprobe. The ^1^H NMR chemical shifts were referenced to the residual DMSO-*d*_6_ (δ 2.49 ppm) and ^13^C chemical shifts are referenced to the DMSO-*d*_6_ solvent peak (δ 39.5 ppm). The low resolution ESI-QIT-MS was recorded on a Bruker-Hewlett Packard 1100 Esquire–LC system mass spectrometer.

### 2.4 Cell culture

The human cell lines HT-29 (ATCC HTB-38), M059K (ATCC CRL-2365), and WI38 (ATCC CCL-75) were obtained from the American Type Culture Collection (ATCC). Cells were cultivated as previously described [14, 15]. HT-29 cells were used for investigation of anti-mitotic natural products because of their capacity to remain in mitotic arrest [16]. HT-29 cells were plated at a density of 3.0 × 10^5^ cells/25 cm^2^ or 1.0 × 10^6^ cells/75 cm^2^ flask and cultured for 48–72 h prior to treatment. M059K and WI38 cells were plated at a density of 1.5 × 10^5^/25 cm^2^ flask and cultured for 24–48 h prior to treatment. The compounds nocodazole (660 μM, Sigma-Aldrich; M1404), paclitaxel (1 mM, Sigma, T7402) and hymenoratin (3.8 mM) were dissolved in DMSO and stored at −20 °C. In not-treated cells, DMSO was added as a solvent vehicle control to 0.3% (volume/volume).

### 2.5 Light microscopy and time-lapse video microscopy

Images of cells in culture were captured with an Infinity 1 camera operated by Infinity Capture imaging software (Lumenera Corporation) on an Olympus CKX41 inverted microscope. Images were processed using Adobe Photoshop (CC 2014.1.0). Rounded cells were counted using Image J software (IJ 1.46r). Time-lapse video microscopy images were collected BioTek Cytation 5 imager operated by Gen5 software. At least 200 HT-29 cells were counted for each treatment.

For time-lapse video microscopy HT-29 cells were plated in 6 well culture plates and incubated at 37 °C for 48 h prior to treatment. Imaging was performed in a Cytation™ 5 Cell Imaging Multi-Mode Reader using Gen5 software (BioTek Instruments, USA) to collect phase contrast images every 10 min for 24 or 48 h post-treatment using the 10x objective in a controlled chamber. Cells were manually scored for a rounded or flat morphology for times up to 48 h. At least 100 cells were counted per treatment.

### 2.6 Cell viability (MTT) assay

Plant extract cytotoxicity was measured by the MTT (3-((4,5)-dimethylthiazol-2-yl)-2,5-diphenyl-tetrazolium) assay (Sigma-Aldrich; M2128-1G) [14]. HT29 cells were plated at 4.0 × 10^5^ cells/96 well culture plate and cultured at 37 °C for 72 h prior to treatment. M059K and WI38 cells were plated at 2.5 × 10^5^ cells/96 well culture plate and cultured at 37 °C for 48 h prior to treatment. All treatments were run in triplicate at 96 h and experiments were performed at least three times. After the specified treatment time, 20 μl of MTT solution (5 mg/ml MTT in phosphate buffered saline (PBS) (137 mM NaCl, 3 mM KCl, 100 mM Na_2_HPO_4_, 18 mM KH_2_PO_4_) was added to the media in each well and the plates were incubated at 37 °C for 3.5 h. The media were then aspirated and 150 μl of MTT solvent (4 mM HCl, 0.1% (v/v) octyl-phenoxy-polyethoxy-ethanol, in isopropanol) was added to each well. Plates were placed on a shaker for 30 min in the dark, and absorbance was measured at 590 nm using an Epoch microplate spectrophotometer (BioTek Instruments, USA) powered by Gen5 software. Results were expressed as IC_50_ concentrations; the half maximal inhibitory concentration of the compound or plant extract that reduced the absorbance of MTT by 50%, by comparison to 0.1% (v/v) DMSO-treated cells. The normalized percent absorbance was calculated (1):

(1) Normalized percent absorbance = (absorbance/DMSO absorbance) × 100

The log concentrations of the compound were plotted against the normalized percent absorbance using Microsoft Excel software. Analysis was performed with GraphPad Prism 5 software, using non-linear regression (log (inhibitor) versus normalized response), to estimate the IC_50_ concentrations. Standard curves were plotted using Eq. (2):

(2) Y = maximum + (maximum – minimum)/ (1 + 10(X-LogIC_50_))

Where Y is the percent cell viability, and maximum is the percentage of viable cells after treatment with 0.1% DMSO, minimum is the percentage of viable cells after treatment with the highest concentration of the genotoxic molecule and X is the log10 value of the treatment concentration.

### 2.7 Immunofluorescence microscopy

HT-29 cells were seeded on glass coverslips in 6 well culture plates and incubated at 37 °C for 48 h prior to treatment. After 24 h treatment, cells were fixed at room temperature for 20 min in 3% (v/v) paraformaldehyde (Fisher Scientific; 30525-89-4), diluted in PBS. Fixation was quenched with 50 mM NH_4_Cl in PBS followed by permeabilization for 5 min using 0.2% (v/v) Triton X-100 in PBS and blocking for 30 min with 3% (w/v) BSA in PBS-T (0.1% (v/v) Tween-20 diluted in PBS). Cells were then incubated at 4 °C overnight with anti-phospho-Ser10 histone H3 (Millipore; 06-570(CH); 1:1000), or anti-α-tubulin (Santa Cruz Biotechnology; sc-53030; 1:200). After washing with PBS-T, cells were incubated for 45 min at room temperature with Alexa Fluor 594 AffiniPure goat anti-rabbit IgG (Jackson ImmunoResearch; 111-585-003; 1:400), Alexa Fluor 488 rabbit anti-rat IgG (ThermoFisher; A11006; 1:400). Nuclei were stained with 300 nM DAPI (4′, 6-diamidino-2-phenylindole) (Fisher; LSD1306) in PBS for 15 min and coverslips were mounted onto microscope slides using ProLong Gold Antifade reagent (ThermoFisher; P36934). Cells were observed and recorded with a Cytation™ 5 Cell Imaging Multi-Mode Reader using Gen5 software (BioTek Instruments, USA). Cells positive for phospho-Ser10 histone H3 and total number of cells (DAPI stained) were counted with Gen5 software. A minimum of 200 cells were counted for each treatment and the mean and standard error of the mean percentage of PH3-positive cells of at least three independent experiments were calculated. For the analysis of mitotic spindles, at least three independent experiments were performed and a minimum of 50 mitotic cells per treatment were observed in total. DMSO-treated mitotic cells were used as a reference for baseline mitotic spindle morphology.

### 2.8 Cell extraction and western blots

HT-29 cells were plated at 1 × 10^6^ cells/75 cm^2^ flask and incubated at 37 °C for 48 h prior to treatment. After treatment, mechanical shake-off was used to collect mitotic cells, which are weakly adherent, and separate them from interphasic cells that are strongly adherent [14, 17]. Interphasic cells were collected by trypsinization. Cell isolation was confirmed by light microscopy. Cells were re-suspended in ice cold lysis buffer (50 mM HEPES, pH 7.4, 50 mM NaF, 10 mM EGTA (ethylene glycol tetraacetic acid), 50 mM β-glycerophosphate, 1 mM ATP, 1 mM DTT (dithiothreitol), 1% Triton X-100 (v/v), 10 μg/mL RNase A (Sigma-Aldrich; R6513-250MG), 0.4 U/mL DNase I (Invitrogen; I354Ba) and protease inhibitor cocktail (Roche; 11836170001)) at a concentration of 20,000 cells/μL, passed through a 26-gauge needle five times and incubated on ice for 30 min. The suspension was centrifuged at 10,000 × g for ten minutes at 4 °C, aliquoted into 1.5 mL microfuge tubes and stored at −80 °C. Extracts were prepared for electrophoresis after being boiled for five minutes in the presence of 2x SDS (sodium dodecyl sulphate) sample buffer (20% (v/v) glycerol, 10% (v/v) DTT, 6% (w/v) SDS, 500 mM Tris, pH 6.8). Western blots were performed as previously described [18] using anti-Cdk1 (Signalway Antibodies; 21236-2; 1:500), anti-phospho-tyr15 Cdk1 (Signalway Antibodies; 11244-2; 1:500), anti-cyclin B1 (Santa Cruz Biotechnology; sc-245; 1:100), anti-actin (Santa Cruz Biotechnology; sc-58673; 1:200), anti-cyclin A (Santa Cruz Biotechnology; sc-271682; 1:500), primary antibodies, and alkaline phosphatase coupled anti-mouse IgG (Promega; PRS3721; 1:2500) or anti-rabbit IgG (Promega; PRS3731; 1:2500) secondary antibodies. Western blot analyses were performed three times.

### 2.9 Statistical analysis

Data were analyzed using Microsoft Excel 2016 and GraphPad Prism 5 software. Data were plotted as means of three independent experiments ± standard error of the mean. One-way analysis of variance (ANOVA) with Tukey's or Dunnett's post hoc test were used to analyze results from light microscopy and immunofluorescence microscopy assays. Differences were considered significant when *p* < 0.05.

## 3 Results

### 3.1 Extracts prepared from *H. richardsonii* induce mitosis in cancer cells

**Figure 1:**
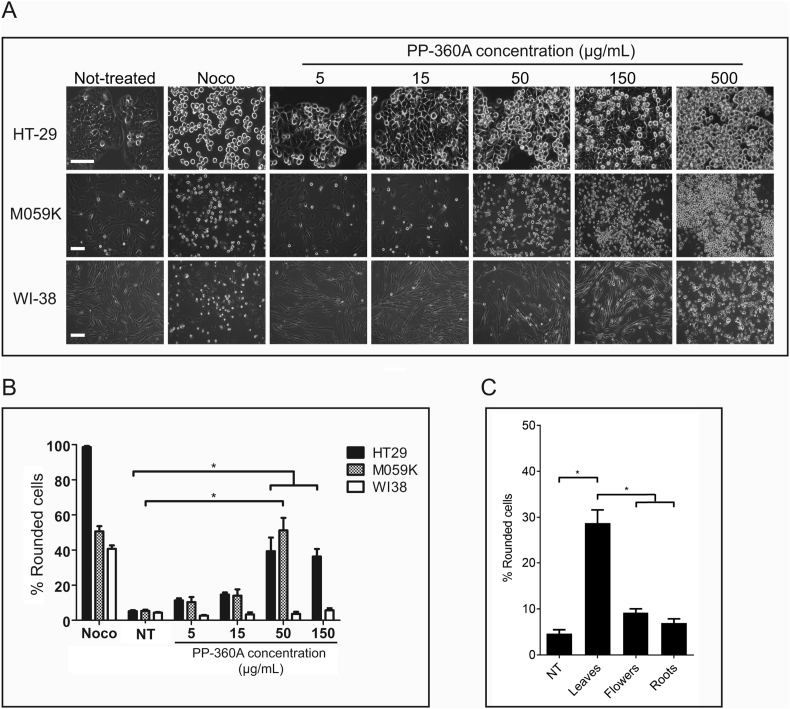
Human cancer cells treated with extracts prepared from *H. richardsonii* leaves acquire a rounded morphology. A. HT-29, M059K, and WI-38 cells were either not-treated or treated with nocodazole (Noco), or treated with *H. richardsonii* PP-360A extract at varying concentrations for 24 h and observed by light microscopy for cells with rounded morphology, indicating mitosis. Scale bars represent 50 μm. B. The mean percentages of rounded cells in a population at 24 h were determined for each treatment described in A (NT, not-treated). The standard errors of the means are shown. Asterisks represent treatments that are significantly different from other treatments (p < 0.05). C. HT-29 cells were either not-treated or treated with 50 μg/mL of extracts prepared from leaves, flowers or roots. Cells were observed by light microscopy and the percentage of rounded cells was calculated. The standard errors of the means are shown. Asterisks represent treatments that are significantly different from other treatments (p < 0.05).

We extracted *H. richardsonii* leaves with either 75% ethanol (v/v) (PP-360A) or with 100% dichloromethane (PP-360B). Three cell lines (HT-29, M059K, WI-38) were either not treated or treated with the tubulin toxin, nocodazole (Noco), or with a range of concentrations of extracts PP-360A (Figure 1). Images of representative cells that were not treated or treated with nocodazole or PP-360A (Figure 1A) are shown. After 24 h the percentage of rounded cells was determined (Figure 1B). Treatment with PP-360A induced nearly 40% rounded cells in HT-29 and 51% in M059K cells at 50 μg/mL concentrations. PP-360B also induced cell rounding in HT-29 cells, but in M059K cells only a maximum of 12.7% of the cells were rounded at any concentration (not shown), therefore, we selected PP-360A for further experiments. Interestingly, the cell rounding activity in normal, diploid WI38 cells was not observed with PP-360A at any concentration tested (Figure 1B). We prepared 75% ethanol (v/v) extracts from *H. richardsonii* leaves, flowers or roots to compare them for cell rounding activity (Figure 1C). Leaves extract induced 28.8 ± 2.9% of rounded cells after 24 h of treatment, which was higher than not-treated cells (4.6 ± 0.8%). Root or flower extracts elicited responses similar to that of not-treated cells: 7.0 ± 0.9% of rounded cells after treatment with the root extract and 9.2 ± 0.9% with the flower extract. These data indicated that PP-360 leaves extract contain a chemical that induces a cell rounding activity that is characteristic of mitotic arrest.

The cytotoxicity of PP-360A was tested in HT-29, M059K, or WI38 cells that were treated with either DMSO only, or with concentrations of extracts ranging from 0.3 to 1000 μg/mL for 96 h. PP-360A had an IC_50_ of 123 ± 16 μg/mL in HT-29 and 56 ± 6 μg/mL in M059K. In the non-cancer cell line WI38, extract PP-360A had an IC_50_ of 96 ± 24 μg/mL, even though it had not induce cell rounding.

**Figure 2:**
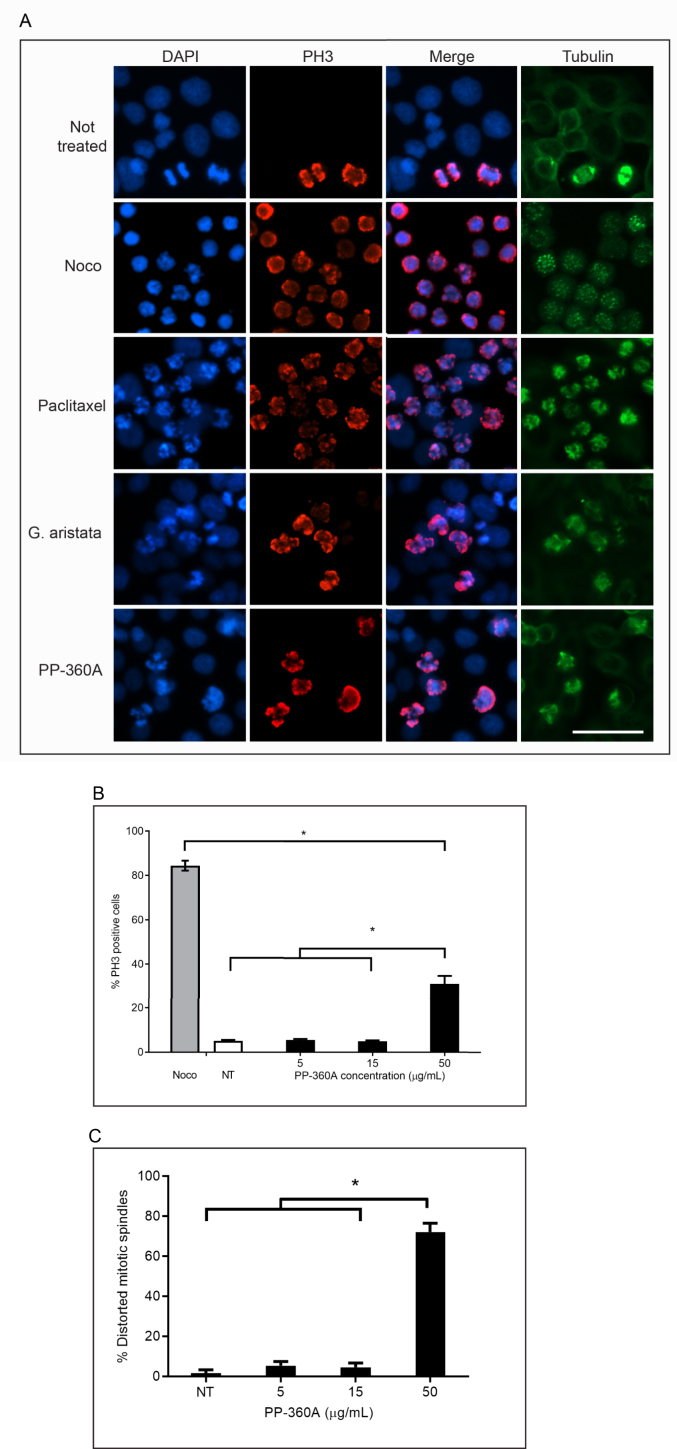
Cells treated with PP-360A extracts acquire phospho-histone H3 and distorted mitotic spindles. A. HT-29 cells were either not-treated or treated with nocodazole (Noco), paclitaxel, *G. aristata* extract or PP-360A for 24 h and stained with DAPI (blue) to detect DNA, phospho-histone H3 antibodies (red) and anti-α-tubulin antibodies (green). The merge column is the combination of DAPI and phospho-histone H3 staining. Cells were analysed by immunofluorescence microscopy and representative images are shown. Scale bar represents 50 μm. B. The mean percentages of cells exhibiting phospho-histone H3 signals after treatment with either nocodazole (Noco) or a range of PP-360A concentrations, or not-treated (NT) as described in A were calculated. Standard errors of the means are shown and treatments that are significantly different from other treatments are marked by an asterisk (p < 0.05). C. The mean percentages of cells exhibiting distorted mitotic spindle after treatment with a range of PP-360A concentrations or not-treated (NT) were calculated. Standard errors of the means are shown and treatments that are significantly different from other treatments are marked by an asterisk (p < 0.05).

To determine if cell rounding induced by PP-360A was caused by an arrest in mitosis, we used immunofluorescence microscopy to detect the mitotic protein phospho-histone H3 (PH3) and tubulin organization (Figure 2A). Cells were either not-treated, or treated with either nocodazole, paclitaxel (Taxol®), *G. aristata* extract (a previously characterized plant from the Asteraceae family) or PP-360A. The mean percentages of PH3-positive cells was 4.5 ± 0.6% for not-treated cells and 83 ± 3.6% for nocodazole and 97 ± 2.6% for paclitaxel treated cells (Figure 2B). By contrast, 31.3 ± 3.6% of the cells treated with PP-360A at 50 μg/mL were positive for PH3, a value that was similar to 37 ± 4.6% of the *G. aristata* extract.

We also observed treated cells after staining with anti-α-tubulin antibodies (Figure 2A). Not-treated cells showed interphasic microtubules and mitotic cells with bipolar shaped spindles. Nocodazole and paclitaxel have opposite effects: nocodazole prevents the formation of the mitotic spindle leaving little or punctate staining, whereas paclitaxel stabilizes spindle fibers and increases their staining intensity. After 24 h of treatment with PP-360A, we observed a striking distortion in the mitotic spindle organization of 72 ± 4% of the mitotic cells (Figure 2C). We concluded that the PP-360A extract prepared from *H. richardsonii* induces a mitotic arrest in human cancer cells.

### 3.2 Isolation and anti-mitotic activity of the sesquiterpene lactone, hymenoratin

**Figure 3:**
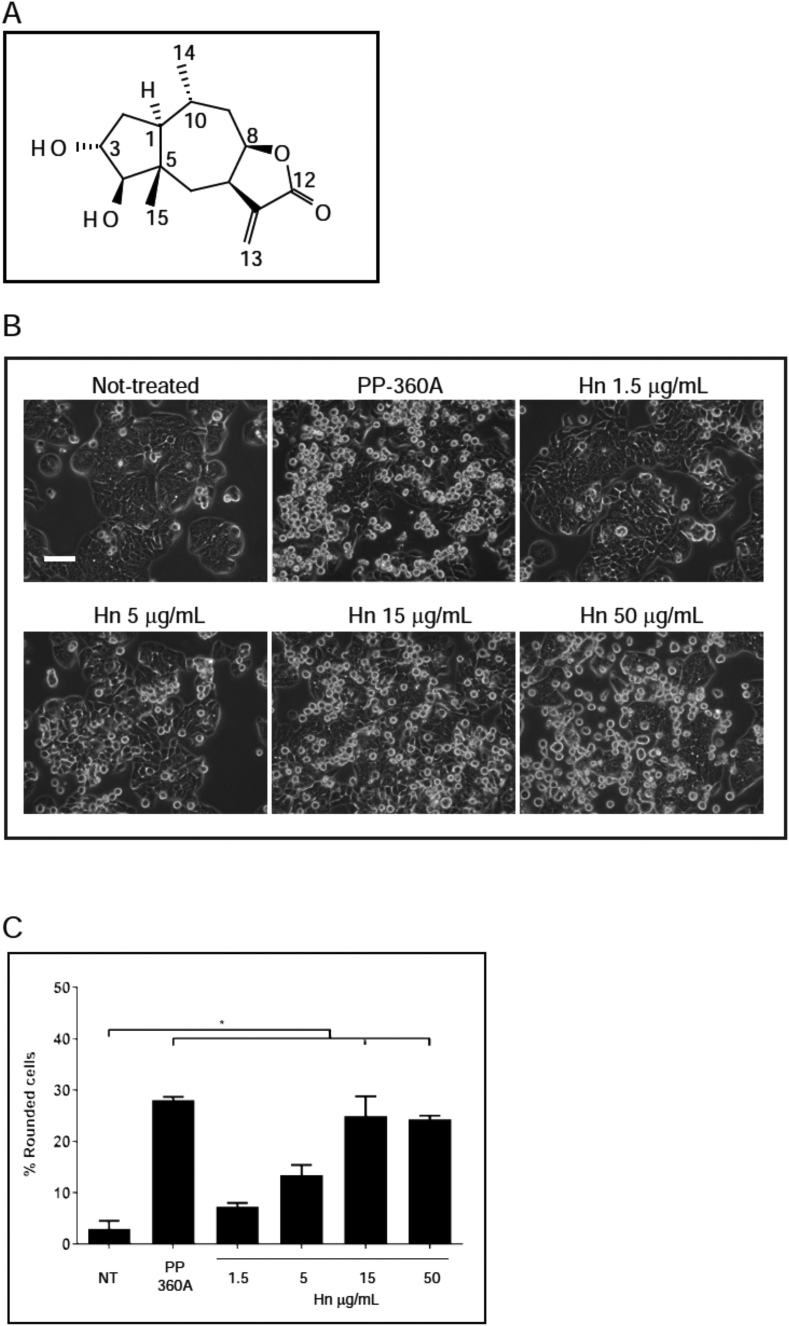
Hymenoratin, isolated from *Hymenoxys richardsonii* by biology guided fractionation induces cell rounding. A. The structure of hymenoratin is shown and carbons are numbered. B. HT-29 cells were either not-treated or treated PP-360A, or with a range of concentrations of hymenoratin (Hn) for 24 h. Representative images taken by phase contrast microscopy are shown. Scale bar represents 100 μm. C. The mean percentages of rounded cells from B were calculated. Standard errors of the means are shown and asterisks show statistical significance (p < 0.05).

We used the cell rounding assay and bioassay guided fractionation to purify the sesquiterpene lactone, hymenoratin (C_15_H_22_O_4_; MW 266 Da), from *H. richardsonii* leaves (Figure 3A). The chemical structure of hymenoratin was established by detailed analysis of 1D and 2D nuclear magnetic resonance (NMR) spectra (Supplemental figures) and comparison of the assigned NMR data to that published in the literature [19, 20, 21]. Comparing Ortega (1968), in which hymenoratin is referred to as odoratin (the name was subsequently changed by Romo [20], and in Gao, 1990 [21] the current ^1^H and ^13^C NMR resonances assigned to the α-methylene-γ-lactone (δ _C_: 169.4, 140.9, 121.9, 77.1, 37.4 ppm; δ_H_: 6.04 (d, *J* = 2.2 Hz, 1H), 5.61 (d, *J* = 1.4 Hz, 1H), 4.81 (m, 1H), 3.19 (m, 1H) ppm), the two secondary alcohols (δ _C_: 84.3 ppm; δ _H_: 3.36 (d, *J* = 6.0 Hz, 1H) ppm and δ _C_: 74.4 ppm; δ _H_: 3.73 (bt, *J* = 7.8 Hz, 1H) ppm), bridge head methyl singlet (δ _C_: 16.1 ppm; δ _H_: 0.66 (s, 3H) ppm) and methyl doublet (δ _C_: 19.7 ppm; δ _H_: 0.91 (d, *J* = 7.0 Hz, 3H) ppm) all compare favourably. The observation of [M + Na]^+^ and [2M + Na]^+^ ions at *m/z* 289.2 and 555.3 Da in the low-resolution electrospray ionization mass spectrum (LRESIMS) of hymenoratin was consistent with the molecular formula C_15_H_22_O_4_ assigned to the compound by the interpretation of the NMR data. Examination of the scalar proton *J* couplings and the TROESY NMR data confirmed the relative stereochemistry to be the same as that established by [19, 21]. The observed [α]^25^_D_ +51° of the current sample of hymenoratin compared favourably to the value measured by Ortega ([α]^25^_D_ +71°) suggesting the same absolute stereochemistry [19].

The cell rounding activity of hymenoratin (Hn) was confirmed in separate experiments (Figure 3B). Cells were either not-treated, or treated with PP-360A, or Hn at a range of concentrations from 1.5 μg/mL to 50 μg/mL, and the percentages of rounded cells induced by each treatment were determined. Not-treated cells had 4.3 ± 0.7% rounded cells and PP-360A treatment induced 28 ± 0.6% rounded cells (Figure 3C). Hn treatment induced a mean value of 7.3 ± 0.8% rounded cells at 1.5 μg/mL, 14 ± 2% at 5 μg/mL, 25 ± 4% at 15 μg/mL, and 24 ± 0.7% at 50 μg/mL. At 150 μg/mL, cells had died.

**Figure 4:**
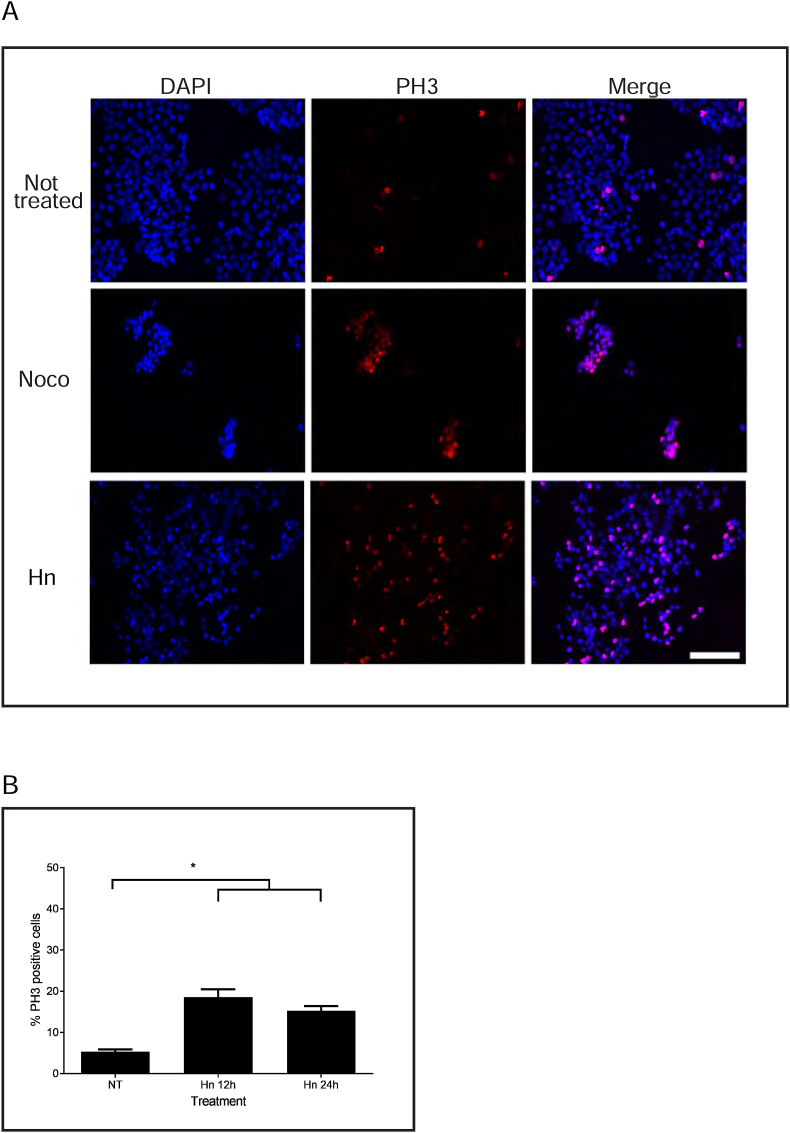
Cells treated with hymenoratin acquire phospho-histone H3 staining. A. HT-29 cells were either not-treated or treated with nocodazole (Noco) or hymenoratin for 24 h and stained with DAPI (blue) to detect DNA or phospho-histone H3 (PH3, red). Cells were analysed by immunofluorescence microscopy, merged, and representative images are shown. Scale bar represents 100 μm. B. The mean percentages of PH3 positive cells from not-treated (NT) and Hn treated cells from A were calculated after 12 and 24 h of treatment. Standard errors of the means are shown and asterisks show statistical significance (p < 0.05).

We tested whether the rounded cells induced by Hn treatment were in mitosis or not (Figure 4). HT-29 cells were either not treated or treated for 24 h with nocodazole or with Hn at 15 μg/mL. Cells were observed by immunofluorescence microscopy using DAPI and PH3 antibodies (Figure 4A). Not-treated cells had about 4% of cells staining with PH3, and Hn induced 19% of PH3 stained cells by 12 h and 15 ± 1% of PH3-positive cells at 24 h (Figure 4B).

**Figure 5:**
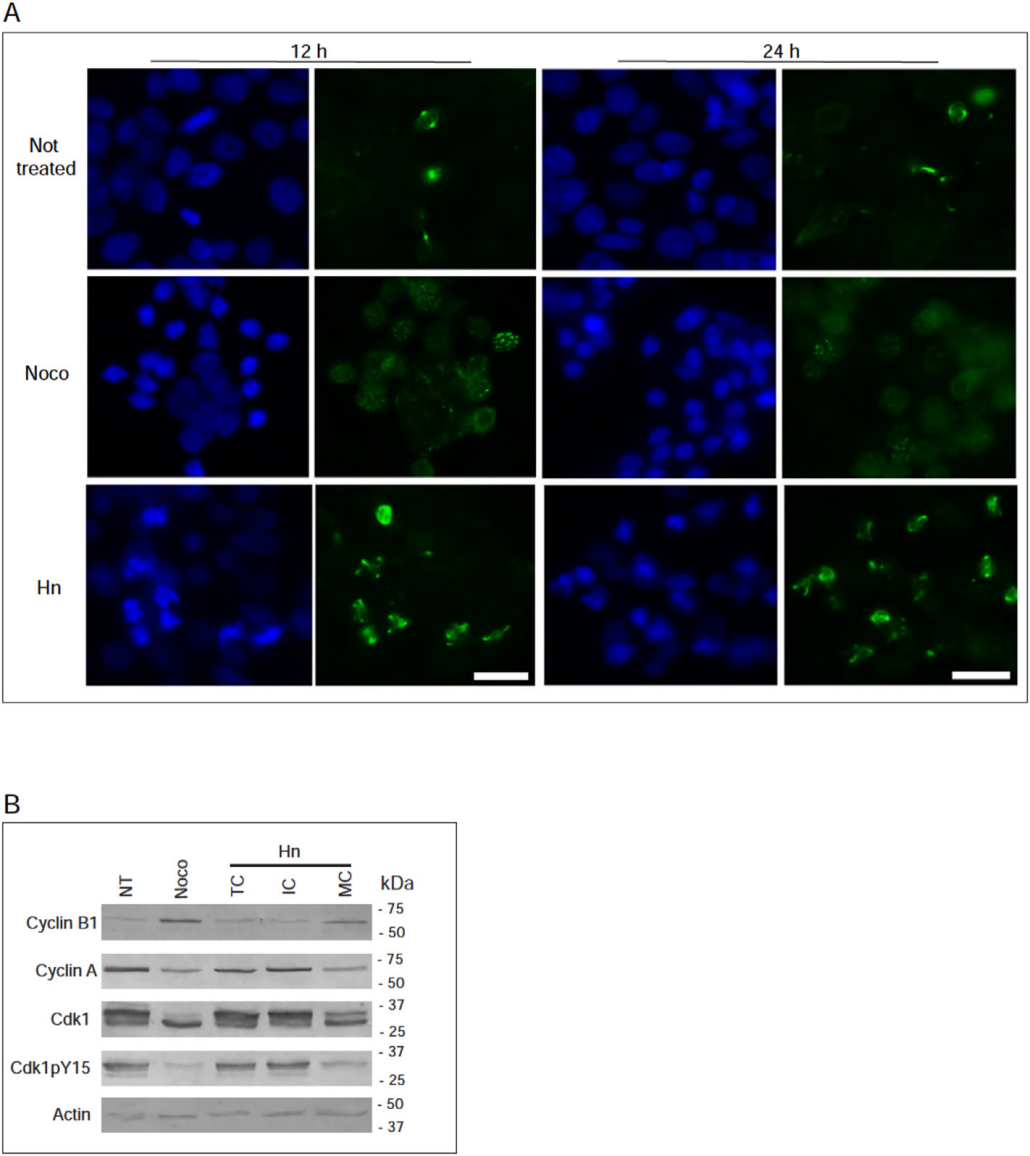
Cells treated with hymenoratin acquire distorted mitotic spindles and contain mitotic proteins. A. HT-29 cells were either not-treated or treated with nocodazole (Noco) or hymenoratin (Hn) for 12 or 24 h and stained with DAPI (blue) to detect DNA or anti-tubulin antibodies (Tubulin, green). Cells were analysed by immunofluorescence microscopy and representative images are shown. Scale bar represents 25 μm. B. HT-29 cells were either not-treated (NT) or treated with nocadazole (Noco) or with hymenoratin (Hn) for 24 h. Mechanical shake-off was used to prepare total cell extracts from all cells (TC), adherent interphasic cells (IC), and rounded mitotic cells (MC). Cell extracts were analysed by western blotting using anti-cyclin B1, anti-cyclin A, anti-Cdk1, anti-phosphoY15 Cdk1, and anti-actin antibodies. Molecular masses are indicated in kDa.

We then tested whether Hn treated cells contained mitotic spindles by immunofluorescence microscopy similar to experiments with which we characterized extract PP-360A (Figure 5A). Not-treated mitotic cells presented normal bipolar spindles, and nocodazole-treated mitotic cells did not form mitotic spindles at 12 or 24 h, as expected. Hn, at 15 μg/mL, induced mitotic spindles in cells at 12 or 24 h, however, the spindles appeared to be distorted in shape at 12 h and at 24 h. To confirm that the Hn treated cells were indeed in mitosis, we tested whether Hn treated cells expressed mitotic proteins (Figure 5B). Cells were either not-treated, or treated with nocodazole, or with Hn. Mitotic cells were collected by mechanical shake-off, giving rise to three samples for testing; total Hn treated cells (TC), Hn treated interphase cells (IC), and Hn arrested mitotic cells (MC). Western blotting analysis revealed that not-treated cells express levels of cyclin B, cyclin A, and pY15-Cdk1 consistent with those of cell with a large interphase component. Nocodazole treated cells had relatively high levels of cyclin B and low levels of pY15-Cdk1. The MC component of Hn treated cells had higher levels of cyclin B than the IC component of treated cells, and the opposite was observed for cyclin A. Like the nocodazole sample, pY15-Cdk1 levels were low in the MC fraction. We conclude that Hn treatment induces mitosis in cancer cells.

## 4 Discussion

This is the first report about the biological activity of hymenoratin, isolated from a plant species found in the prairie ecological zone. There are over 5000 described sesquiterpene structures, and many are known to be synthesized by species of the Asteraceae taxonomical family, of which *H. richardsonii* is a member. Of the 5000 members of this chemical family, fewer than 20 (0.5%) have been reported to have anti-mitotic activity. Our report that hymenoratin arrests cancer cells in mitosis consolidates a growing body of evidence that a subclass of sesquiterpene lactones have anti-mitotic activities [3].

Plants are important sources of anti-mitotic natural products. Several of the most studied anti-mitotic chemicals from plants bind to tubulin, such as taxanes, vinca alkaloids, and colchicine [22]. Sesquiterpene lactones that affect mitosis in human cells are reported to interact with enzymes that regulate mitosis such as Skp1-Cullin-F-box protein (SCF) complex [5], Ran-binding protein 2 protein [23], or tubulin carboxypeptidase [7] rather than tubulin. Sesquiterpene lactone activity is generally reported as cytotoxic, with few examples identified as having anti-mitotic activity [3]. The toxicity of some species of the *Hymenoxys* genus, including *H. richardsonii*, were first identified as toxic to livestock [24, 25]. The sesquiterpene lactones of the types guaianolides, pseudoguaianolides, and modified pseudoguaianolides are considered characteristic of the genus *Hymenoxys* [26]. Examples of these are the sesquiterpene lactones hymenoxynin, hymenolide, paucin in *H. odorata*; anthemoidin, themoidin, vermerin in *H. anthemoides*; floribundin in *H. greenei* and *H. anthemoides* [27]; hymenovin in *H. odorata* and *Dugaldia hoopesi* [28]; and biennin A, 3 and 4-methyl hymenoxon in *H. subintegra* [29]. Specifically in *H. richardsonii*, floribundin and vermerin [27], hymenovin [30] and hymenoratin, hymenograndin, hymenolide, isohymenoloide, 2α-tiglinoyloxydugaldiolide [29] have been described. None of these have been reported to have anti-mitotic activity. We were able to identify an anti-mitotic activity because we applied a phenotypic approach to characterize plant extracts [31, 32].

A characteristic of the treatment with extracts or hymenoratin was that the percentage of mitotic cells ranged from 25 to 35% in contrast to the higher percentage following treatment with nocodazole or paclitaxel. Preliminary observations suggest that the cells either die in mitosis or eventually exit mitosis. The frequency of mitotic arrest and the organization of the mitotic spindle when compared to paclitaxel or nocodazole treatment suggest that extract PP-360A and hymenoratin affect cells by a different mechanism than these two chemicals. Other anti-mitotic sesquiterpene lactones such as 6-O-angeloylplenolin and psilostachyin A and C arrest cells in mitosis with aberrant mitotic spindles and have non-tubulin targets [5, 23], or have non-tubulin but spindle related targets [33].

Hymenoratin is a sesquiterpene lactone of the pseudoguaianolide type, with the α-methylene-γ-lactone at C-8 and several hydroxyl groups, at C-3 and C-4. It has been demonstrated in other studies that the α-β-unsaturated carbonyl group present in the lactone moiety is required for biological activity; for example, the anti-mitotic activities of psilostachyin A and C, as well as that of coronopilin are dependent on this functional group [6, 34]. A Michael-type adduct can form between the α-β-unsaturated carbonyl and cysteines of key enzymes. One might have anticipated that Michael acceptors would target enzymes indiscriminately because 90% of the proteins in human cells have cysteines [35]. Strikingly, a subset of sesquiterpene lactones, such as hymenoratin arrest cells in mitosis rather than causing non-specific toxicity.

Other characteristics of sesquiterpene lactones might influence their selectivity to a particular target [36, 37]. Of the sesquiterpene lactones that arrest cells in mitosis, pseudoguaianolides are the most common type, independent of the lactonization site (i.e. C-6 or C-8). Hymenoratin, 6-O-angeloylplenolin, the psilostachyins A and C, coronopilin, and dehydroleucodine [38] are members of this type. Hymenoratin does not contain lateral chains or secondary alkylating groups; nonetheless, hydroxyl groups present in the molecule, as well as its *cis* conformation might increase the biological activity [39]. Hydroxyl groups may form noncovalent interactions with amino-acids and a *cis* conformation is more flexible than the *trans* counterpart. It is likely that the non-lactone component of the sesquiterpene lactones may have a determinant role in the interaction with the target protein(s). We propose that in future studies natural products like hymenoratin be used as chemical probes to dissect the range of proteins targeted by sesquiterpene lactones during mitosis. In addition, understanding the structure-activity relationship between sesquiterpene lactones and their anti-mitotic effects might lead to the development of novel cancer agents [40].

## 5 Conclusions

Leaf extracts prepared from the toxic Canadian prairie plant, *Hymenoxys richardsonii,* induce cell rounding, mitotic spindles, and phosphohistone H3 staining when applied to colonic or neural human cancer cell lines but not to a normal, non-transformed cell line. By bio-assay guided fractionation the sesquiterpene lactone, hymenoratin was identified as the source of the anti-mitotic activity. Hymenoratin induces a mitotic arrest in treated cells as shown by phosphohistone H3 staining and the presence of mitotic spindles. This is the first report of the anti-mitotic activity of hymenoratin. There is a growing body of evidence that a limited number of sesquiterpene lactone chemical family members have anti-mitotic activity.

## Declarations

### Author contribution statement

Layla Molina: Conceived and designed the experiments; Performed the experiments; Analyzed and interpreted the data; Wrote the paper.

David E. Williams: Conceived and designed the experiments; Performed the experiments; Analyzed and interpreted the data.

Raymond J. Andersen: Conceived and designed the experiments; Analyzed and interpreted the data.

Roy M. Golsteyn: Conceived and designed the experiments; Analyzed and interpreted the data; Wrote the paper.

### Funding statement

This work was supported by the Canada Foundation for Innovation [grant number 34542], The Power Corporation of Canada Dr J. Coutts Donation, the AGILITY Cor Van Raay Innovation Fund [Grant number 2018], and Natural Sciences and Engineering Council of Canada (10.13039/501100000038NSERC) Discovery Grant [Grant numberRGPIN-2017-04398].

### Data availability statement

Data included in article/supplementary material/referenced in article.

### Declaration of interests statement

The authors declare no conflict of interest.

### Additional information

No additional information is available for this paper.

## Appendix A Supplementary data

Molina Heliyon Supp Figures.pdf Molina Heliyon Supp Figures.pdf
