# Supplementary material for: Isolation of a natural product with anti-mitotic activity from a toxic Canadian prairie plant
Source: Heliyon. 2021 May 24;7(5):e07131. doi: 10.1016/j.heliyon.2021.e07131 (PMC8167235; doi:10.1016/j.heliyon.2021.e07131)

Supplemental Figure 1 Molina

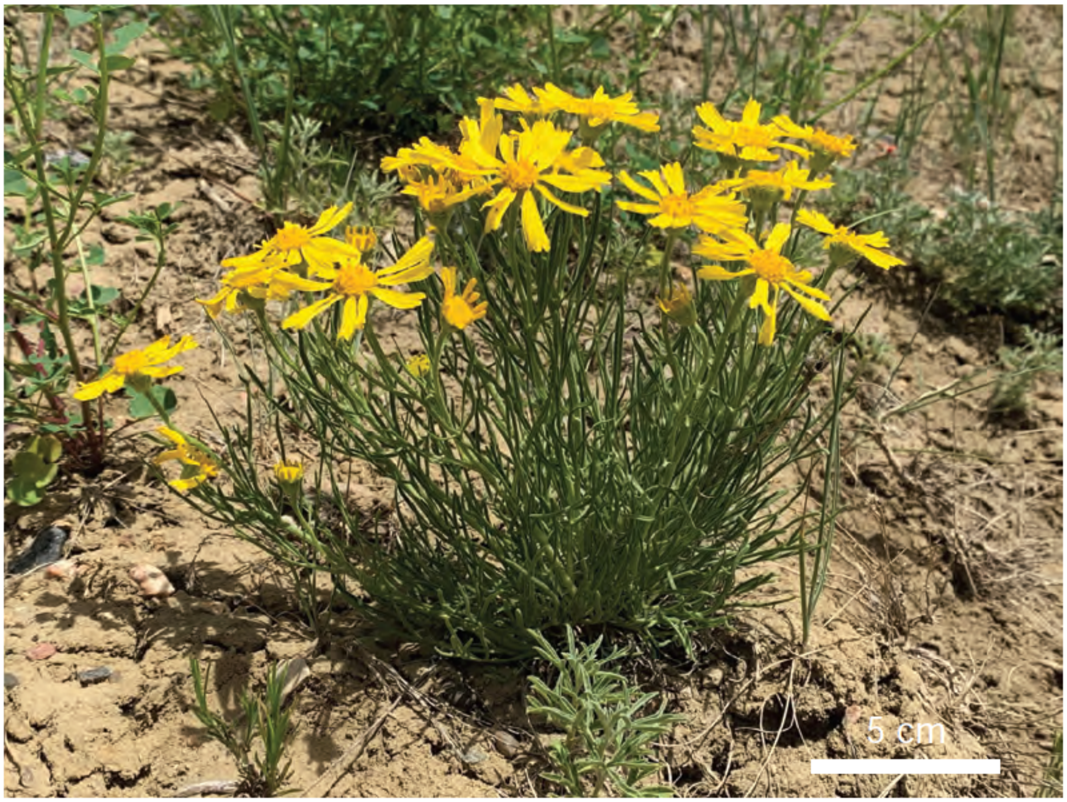

^{1}H NMR Spectrum of Hymenoratin Recorded at 600 MHz in DMSO-d_6

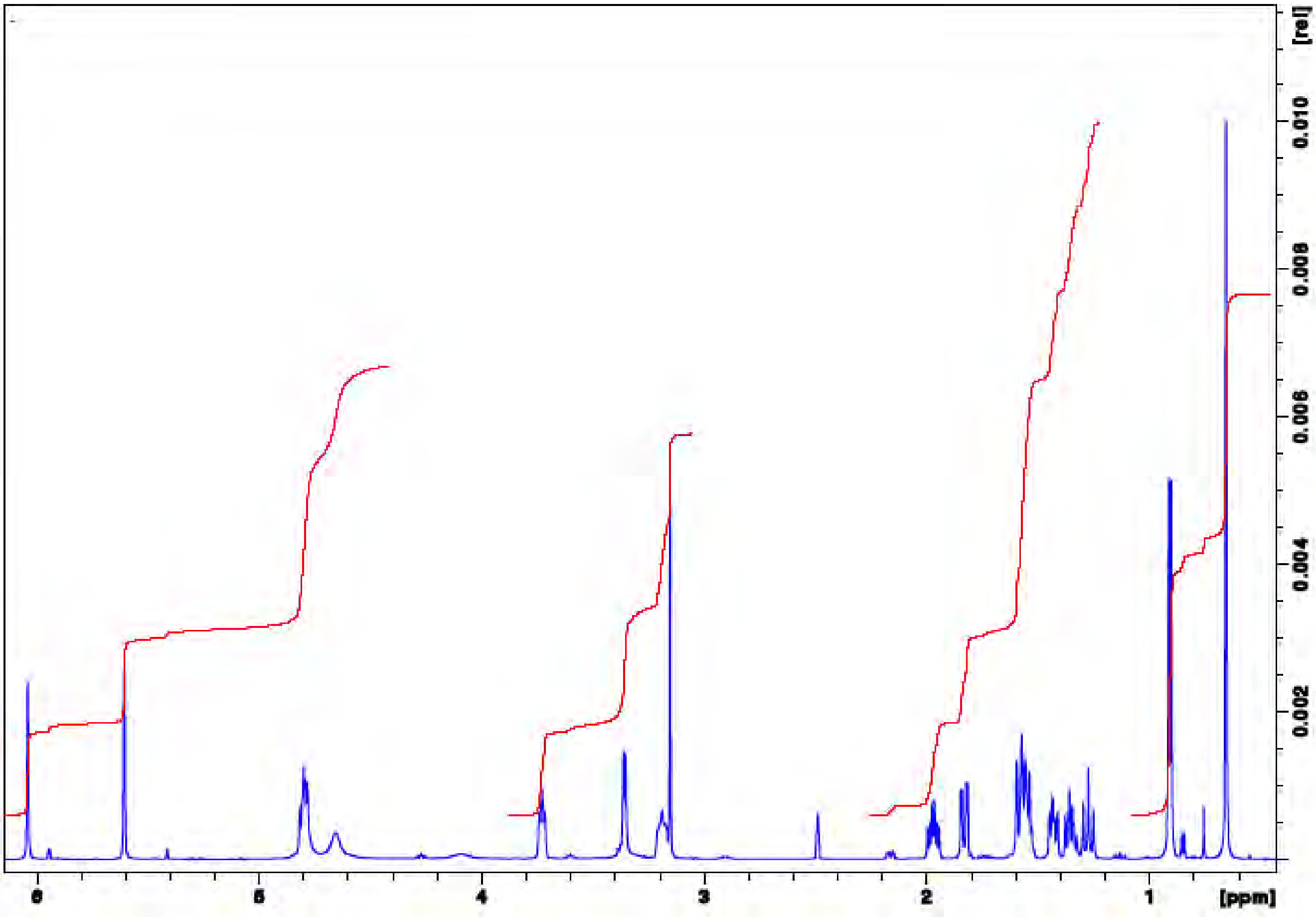

^{13}C NMR Spectrum of Hymenoratin Recorded at 150 MHz in DMSO-d_6

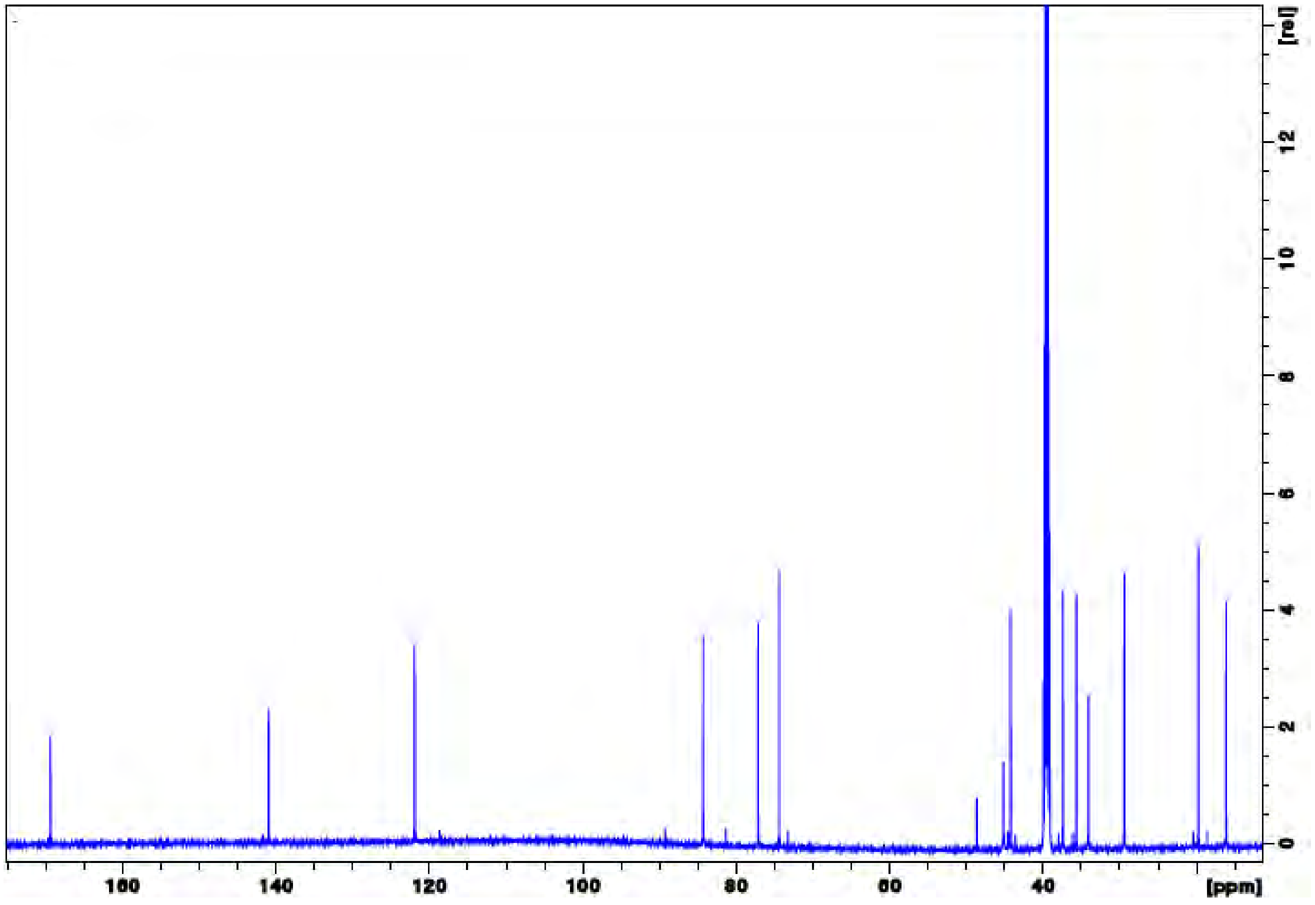

gradCOSY60 NMR Spectrum of Hymenoratin Recorded at 600 MHz in DMSO-d_6

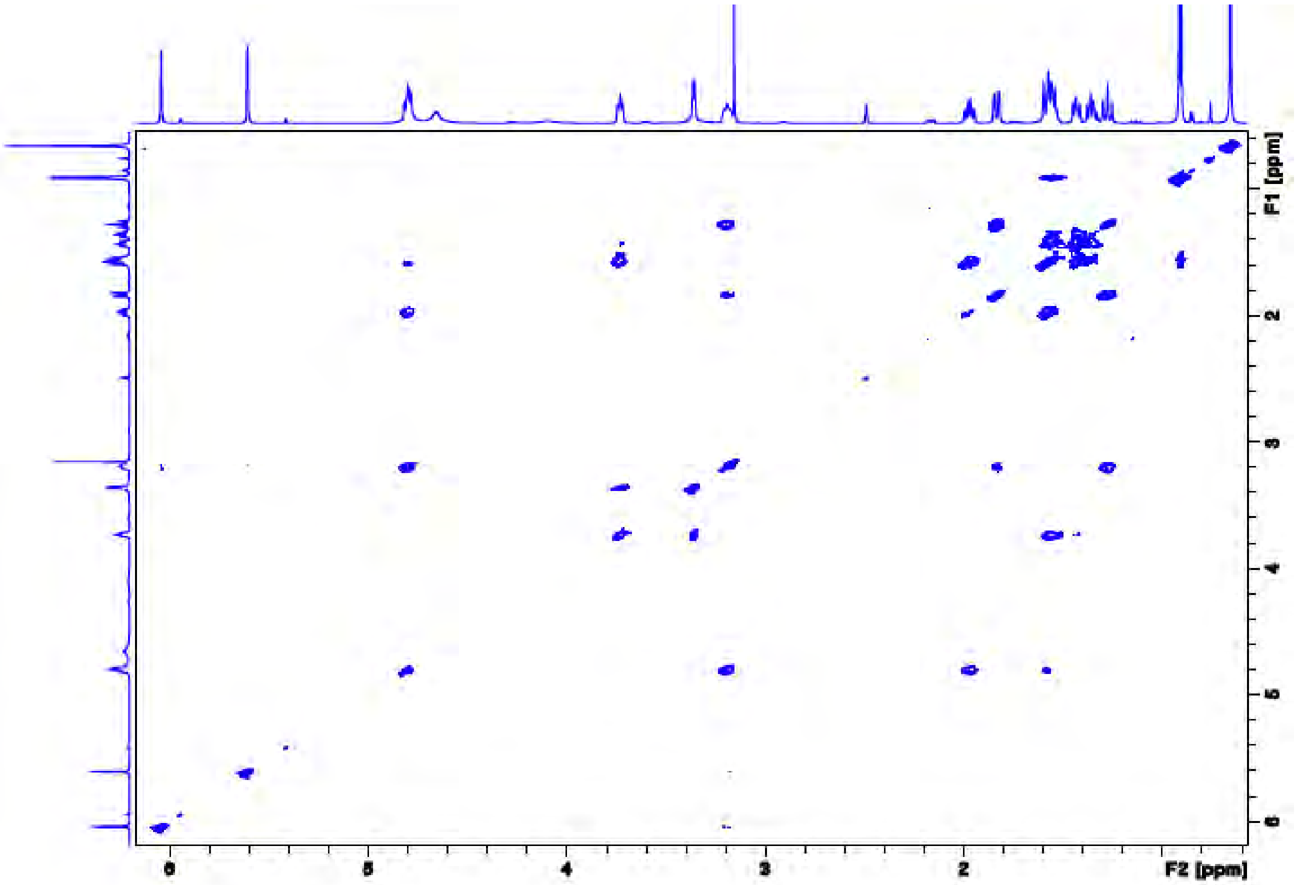

gradHSQC NMR Spectrum of Hymenoratin Recorded at 600 MHz in DMSO-d_6

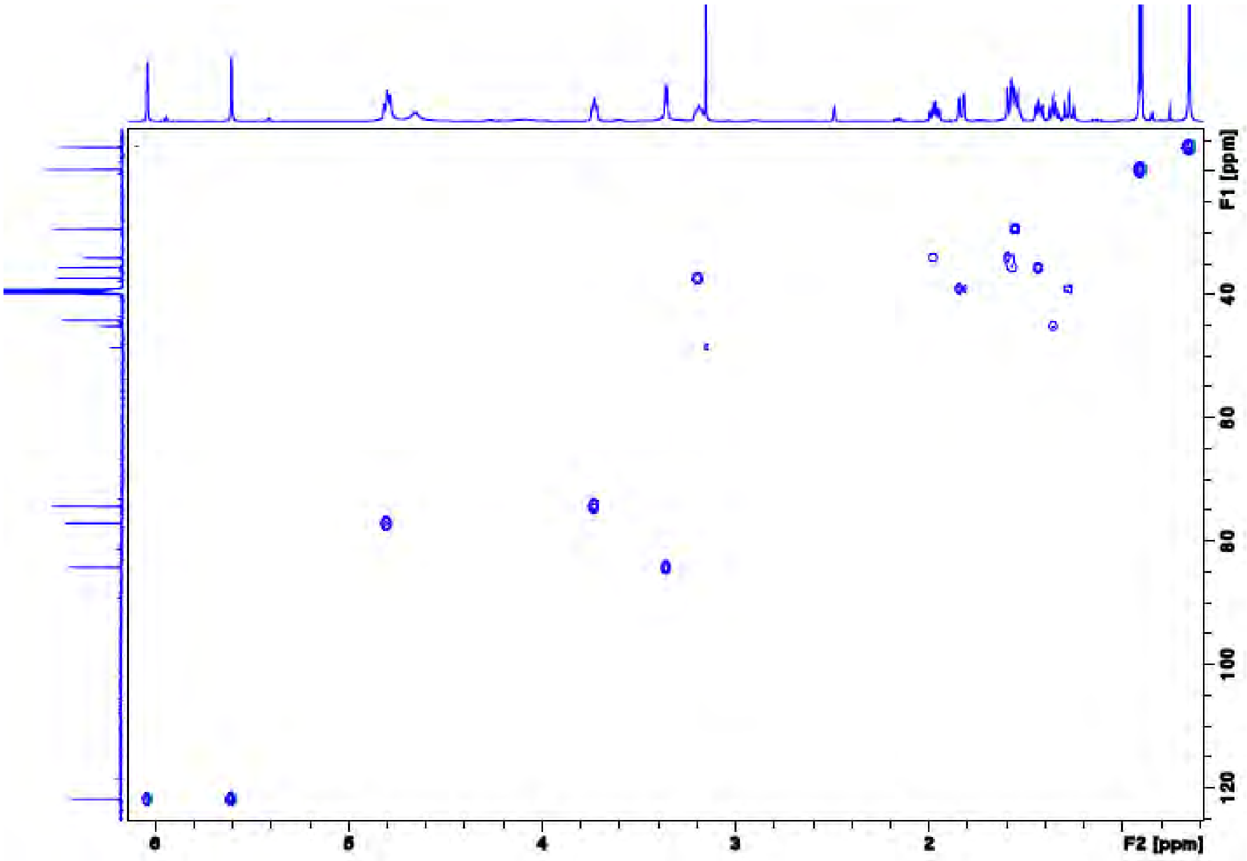

gradHMBC NMR Spectrum of Hymenoratin Recorded at 600 MHz in DMSO-d_6

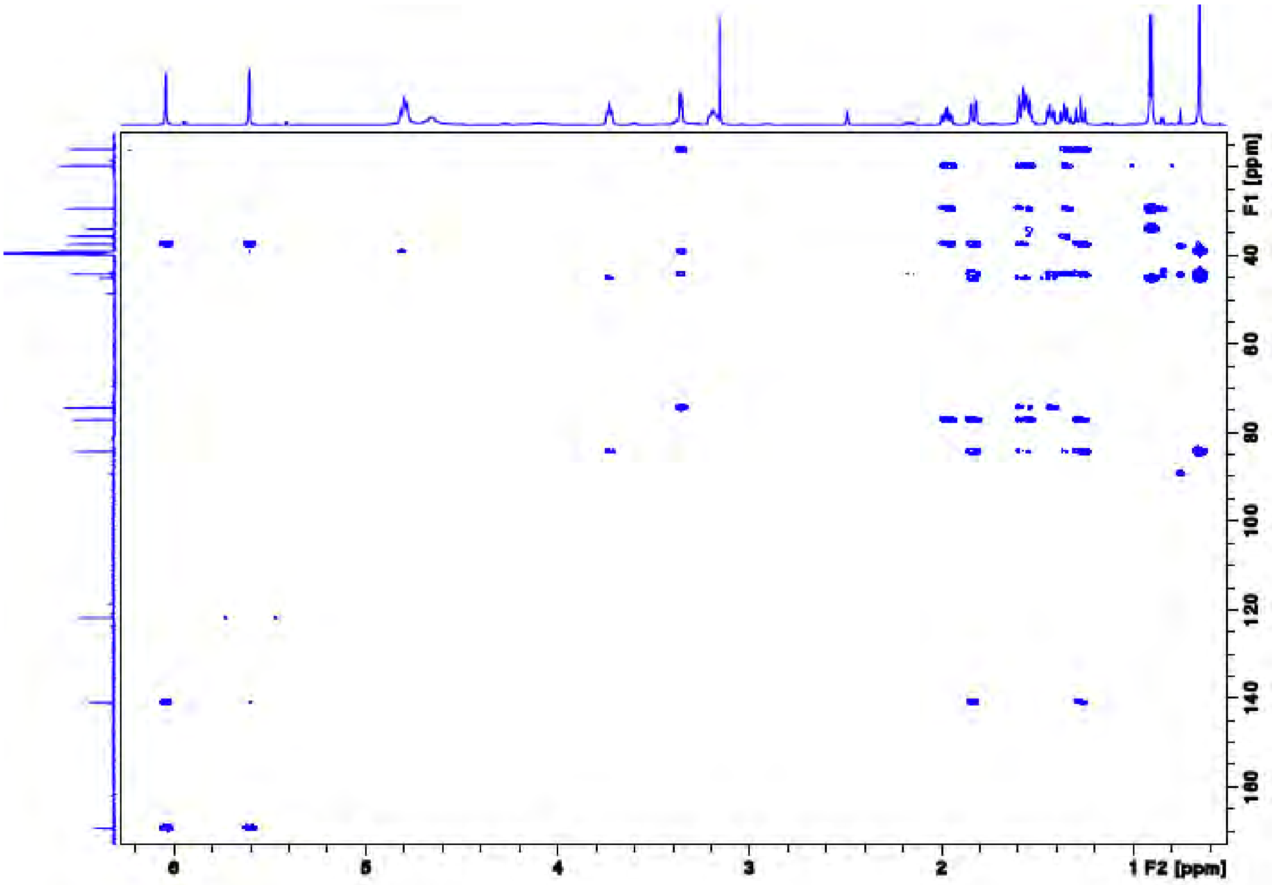

tROESY NMR Spectrum of Hymenoratin Recorded at 600 MHz in DMSO-d_6

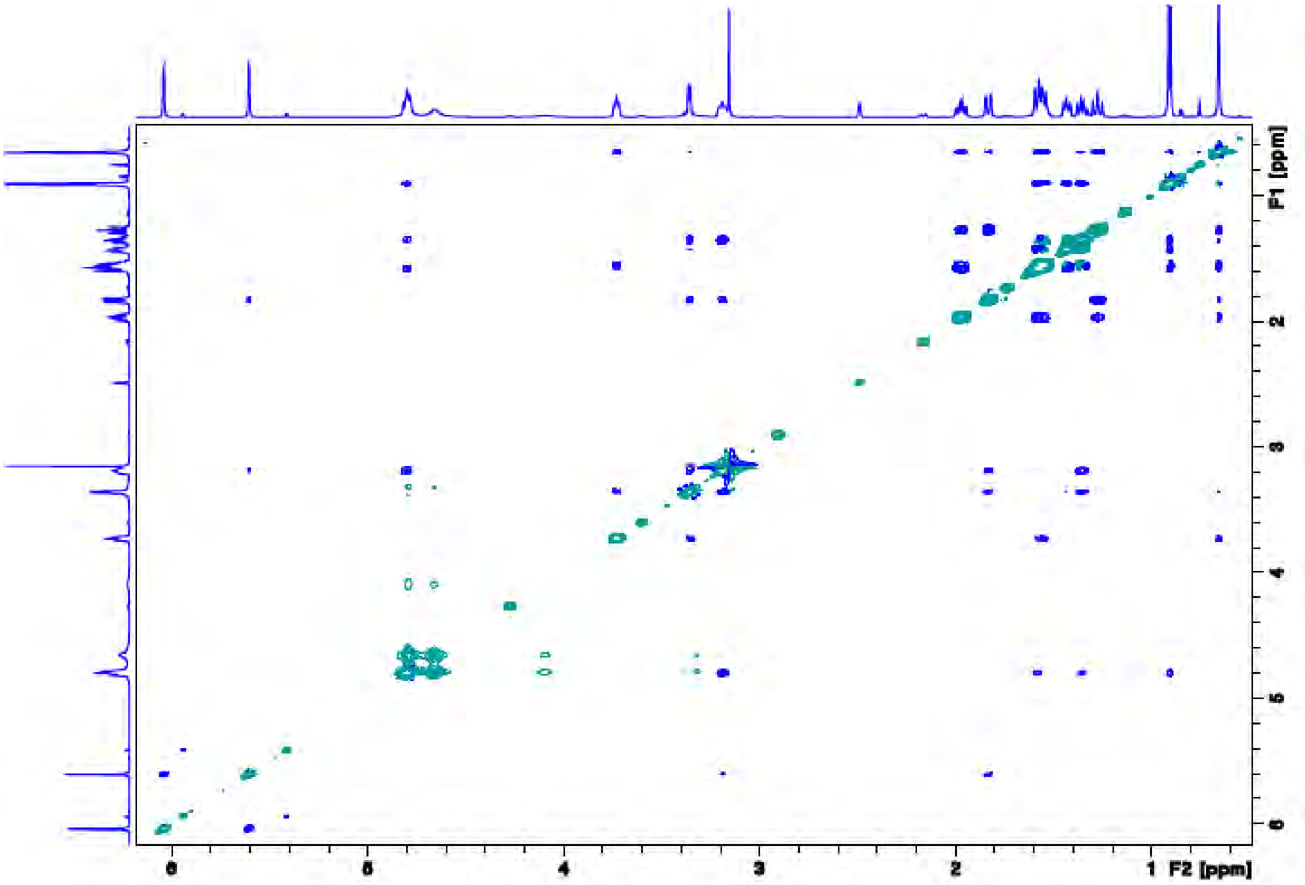

LRESIMS of Hymenoratin

Analysis Name	D:\Data\Mass Spec\Jenny.FI\EL13104_P.d		
Method	fia_400_p_9806.m	Operator	Jenny
Sample Name	EL13104_P	Instrument	HCTultra PTM Discovery System
Comment	HR:EtOAc-C-1, FIA		

Acquisition Parameter

Ion Source Type	ESI	Ion Polarity	Positive	Alternating Ion Polarity	off
Mass Range Mode	Std/Enhanced	Scan Begin	50 m/z	Scan End	1000 m/z
Capillary Exit	121.0 Volt	Skimmer	40.0 Volt	Trap Drive	48.3
Accumulation Time	445 µs	Averages	5 Spectra	Auto MS/MS	off

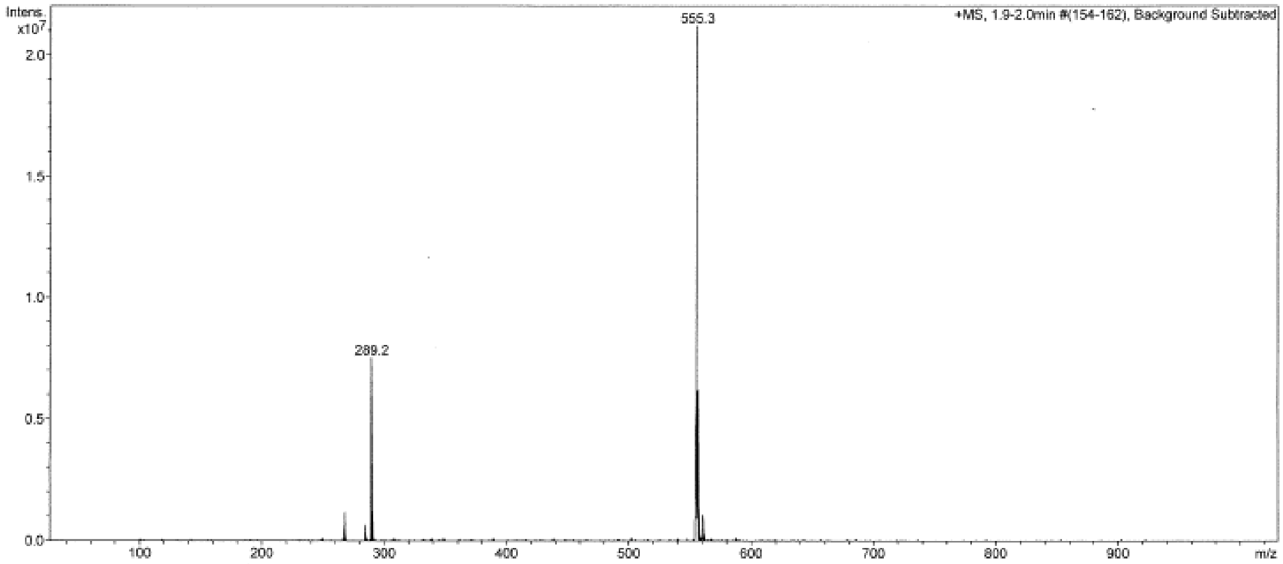

Supplement: Molina Heliyon Supp Figures.pdf [file mmc1.pdf]
